# Supplementary material for: The splicing factor PTBP1 interacts with RUNX1 and is required for leukemia cell survival
Source: Leukemia. 2025 Nov 10;40(1):138–51. doi: 10.1038/s41375-025-02799-w (PMC12789033; doi:10.1038/s41375-025-02799-w)

## Supplementary Figure Legends

**Supplementary Table 1:** Table of antibodies used, with manufacturer, catalog number, and experimental use.

**Supplementary Figure 1**. A) Confocal images of PLAs in REH cells treated with vehicle or 5µM entinostat for 48 hours. B) Graph quantifying PLA signal per field (minimum of 50 cells). C) Representative western blots from HEK293T cells transfected with expression constructs for the indicated proteins, at the indicated ratios of PTBP1 to RUNX1, immunoprecipitated (IP'd) and probed with the indicated antibodies. N=3 for each experiment. **** = p < 0.0001. Scale bar - 20µm.

**Supplementary Figure 2.** A. Representative confocal images and B) graph of PLA intensity/field (minimum of 50 cells) in mouse *CM*$^{+}$ leukemia cells and lineage depleted (lin-) healthy bone marrow cells. Each data point signifies a separate field. C) Representative WB and D) graph of relative PTBP1 levels standardized to actin from 7 independent *CM*$^{+}$ mouse leukemia cells. E) Representative images (10x magnification) and F) graph of colony numbers from colony assays of PTBP1 high and low *CM*$^{+}$ mouse leukemia cells from (A). G) Representative confocal images and H) graph of PLA intensity/nuclei (minimum of 50 cells) in sorted mouse *CM*$^{+}$, KIT+ leukemia stem cells (LSCs) and CSF2RB/CD131+ non-LSCs. N=3 for all experiments. For panel F, N=3 mouse samples, with 3 technical replicates each. **** = p < 0.0001. Scale bar - 20µm.

**Supplementary figure 3.** A) Heatmap and B) profile of all ChIP-seq, CUT&RUN, and CUT&Tag signals over Transcription Start Sites (TSSs) (± 2kb) noted as overlapping with any PTBP1 or RUNX1 peaks. C) Spearman correlation plot of signal over TSS overlapping with PTBP1 or RUNX1 peaks +/-500bp. D) CUT&Tag (PTBP1, RUNX1), CUT&RUN (H3K27me3), and ChIP-seq (Pol2, H3K4me1, H3K27ac, H3K4me3, H3K9ac) signals at Pyruvate Kinase M1/2 (PKM) gene locus.

**Supplementary figure 4.** A) Schematic depicting the *PTBP1* shRNA construct. B) Representative WB and quantification of PTBP1 levels normalized to actin in 3 human AML cell lines transduced with the sh*SCR* and sh*PTBP1*.

**Supplementary figure 5.** A) Heatmap and B) profile showing binding of PTBP1, RUNX1, RNA Pol II and histone modifications at Transcription Start Sites (TSSs) of 83 isoform switched genes. C) Figure displaying counts of 83 isoform switched genes that overlap with PTBP1 eClip peaks. D) PTBP1 eCLIP peak signal and E) Quantification of eCLIP peaks over 83 isoform switched genes compared to 83 randomly selected genes.

**Supplementary figure 6.** A) Integrated Genomics Viewer (IGV) tracks demonstrating CUT&Tag (PTBP1, RUNX1), CUT&RUN (H3K27me3), and ChIP-seq (POL2, H3K4me1, H3K27ac, H3K4me3, H3K9ac) signals over Hexokinase-2 (*HK2*) locus. B) IGV tracks showing CUT&Tag (PTBP1 and RUNX1) signal at Glucose transporter-1 (*SLC2A1*) locus.

**Supplementary figure 7.** A) Representative flow staining of mouse *CM*$^{+}$ leukemia cells treated with 5 µM Entinostat (ENT) or DMSO for 24 hours, stained for KIT to identify leukemia stem cells (LSCs) and CSF2RB to identify non-LSCs and analyzed for glucose uptake. B. Graph showing quantification of mean fluorescence intensity (MFI) of glucose uptake in LSCs and non-LSCs treated as in A. N=3. * p ≤ 0.05. ** = p < 0.01.

## Supplementary Table 1

| Antibody | Manufacturer | Catalog number | Use |
|---|---|---|---|
| GLUT1 | Invitrogen | MA5-43799 | WB |
| ID1 | Biocheck Inc | BCH-1/195-14 | WB |
| PTBP1 | Invitrogen | 32-4800 | WB, IP, IF, PLA, CUT&Tag, CUT&RUN |
| MYC | CST | 2040S | WB |
| MYC-TAG | Invitrogen | MA1-21316 | IP, WB |
| FLAG | SIGMA | A8592 | WB |
| FLAG Magnetic Beads | Sigma-Aldrich | M8823 | IP |
| ACTIN | Santa Cruz | SC47778 | WB |
| GAPDH | Abcam | AB59164 | WB |
| RUNX1 | Abcam | AB23980 | WB, IP [Mass Spec] |
| Hexokinase 2 | CST | 2867S | WB |
| RUNX1 | Invitrogen | PA5-19638 | WB, IP, IF, PLA, CUT&Tag, CUT&RUN |
| H3K27ME3 | CST | 9733S | CUT&Tag |
| Rabbit IgG control | CST | 3900S | IP |
| Guinea pig anti-rabbit | Antibodies Online | ABIN101961 | CUT&Tag, CUT&RUN |
| Rabbit anti-mouse | Abcam | AB46540 | CUT&Tag, CUT&RUN |
| Anti-Mouse | Vector Laboratory | PI-2000 | WB-Secondary |
| Anti-Rabbit | Vector Laboratory | PI-1000 | WB-Secondary |
| Alexa Fluor- 488 | Invitrogen | A-11008 | IF-Secondary |
| Alexa Fluor- 568 | Invitrogen | A-11004 | IF-Secondary |

| | | | |
|---|---|---|---|
| DAPI | BD Biosciences | 564907 | FACS |
| Cy5 Annexin V | BD Biosciences | 559934 | FACS |
| PE-CY7 CD15 | Biolegend | 323030 | FACS |
| BUV395 CD11b | BD Biosciences | 565976 | FACS |
| FITC CD271 (△LNGFR) | Biolegend | 345104 | FACS |
| mCherry BrightComp eBeads | Invitrogen | A54743 | FACS |

Supplementary fig 1

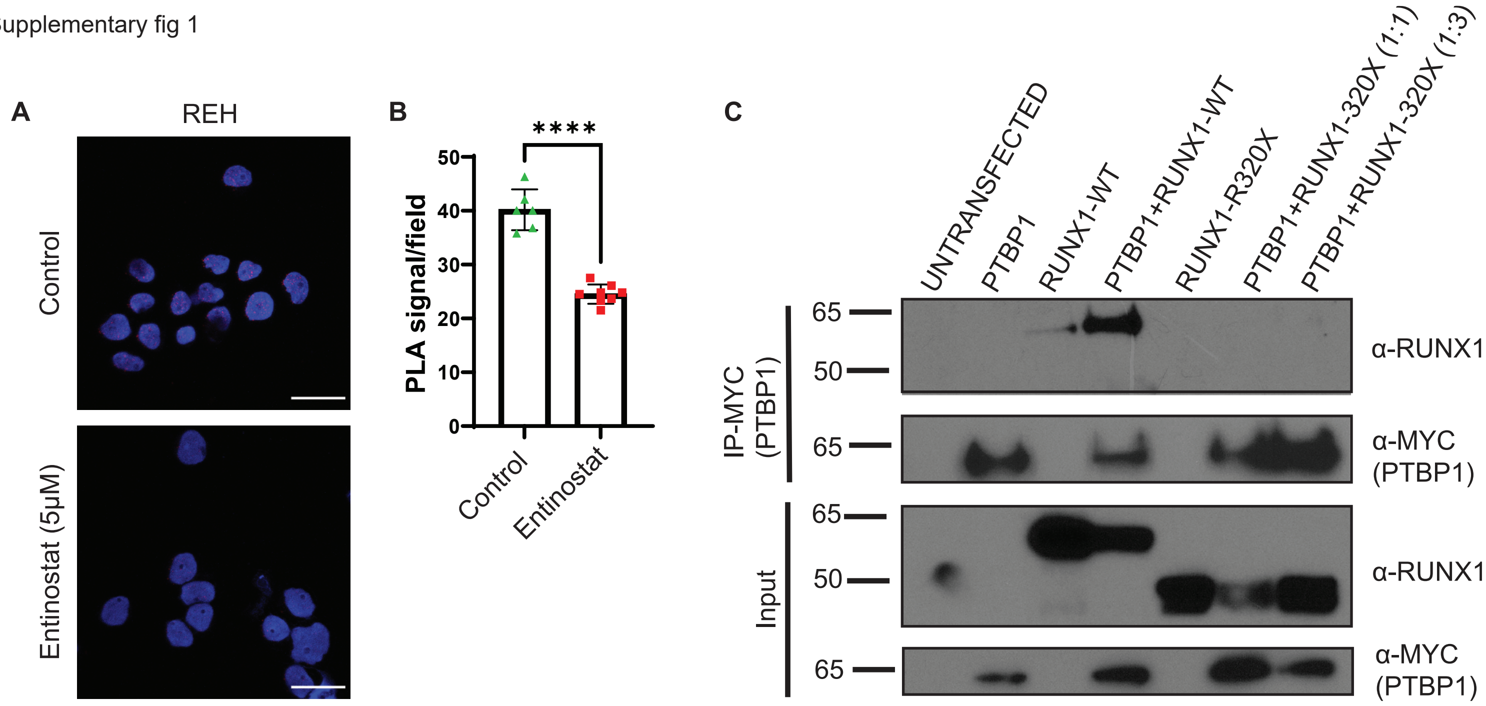

Supplementary fig 2

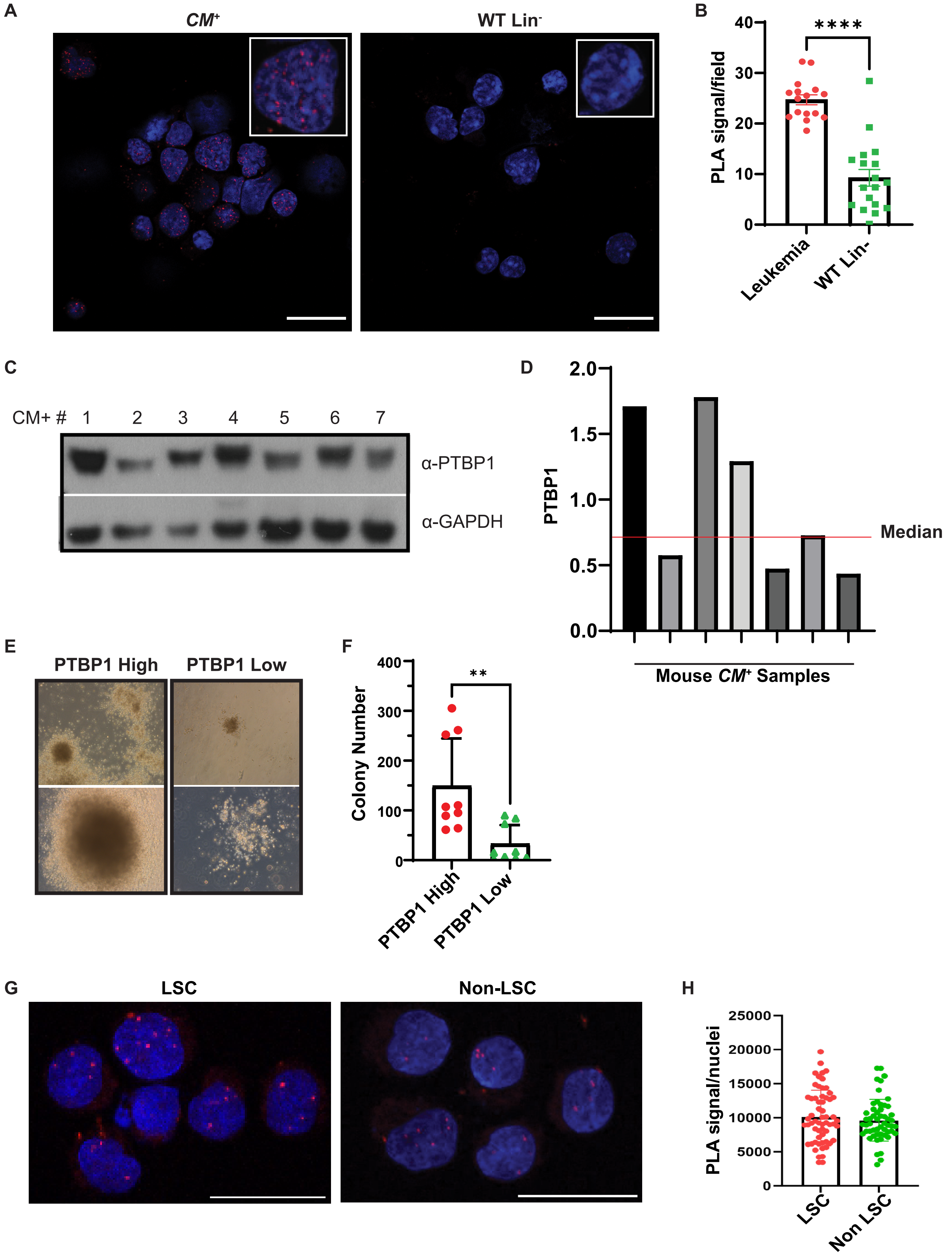

Supplementary fig 3

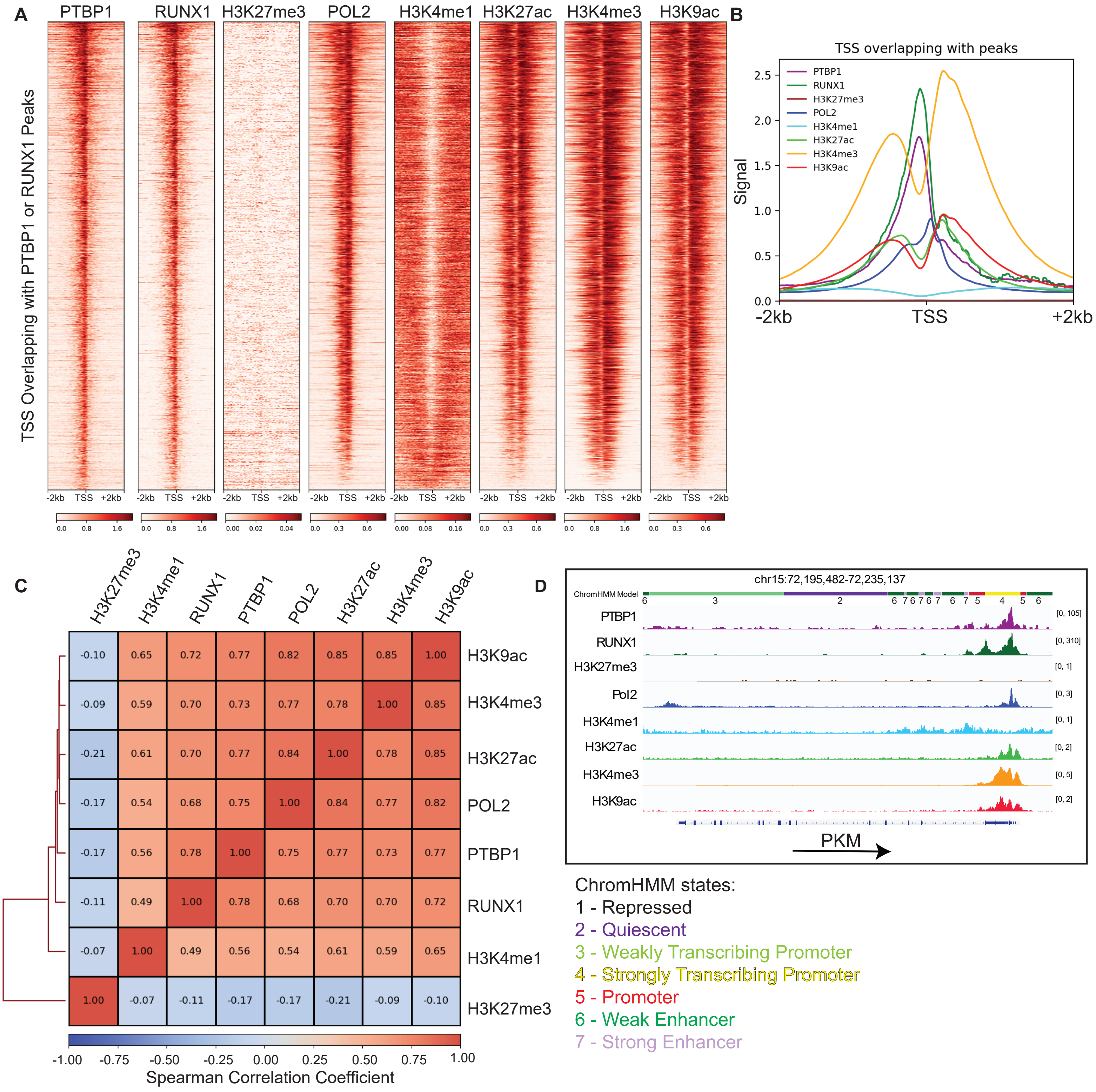

Supplementary fig 4

A

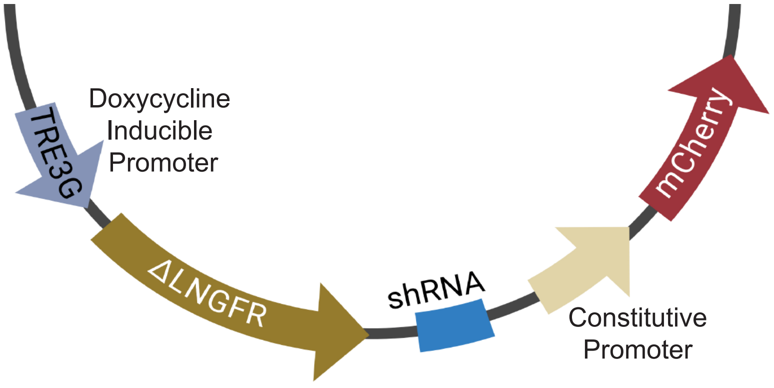


B

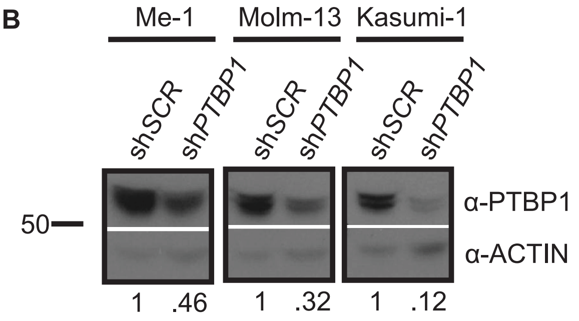

Supplementary fig 5

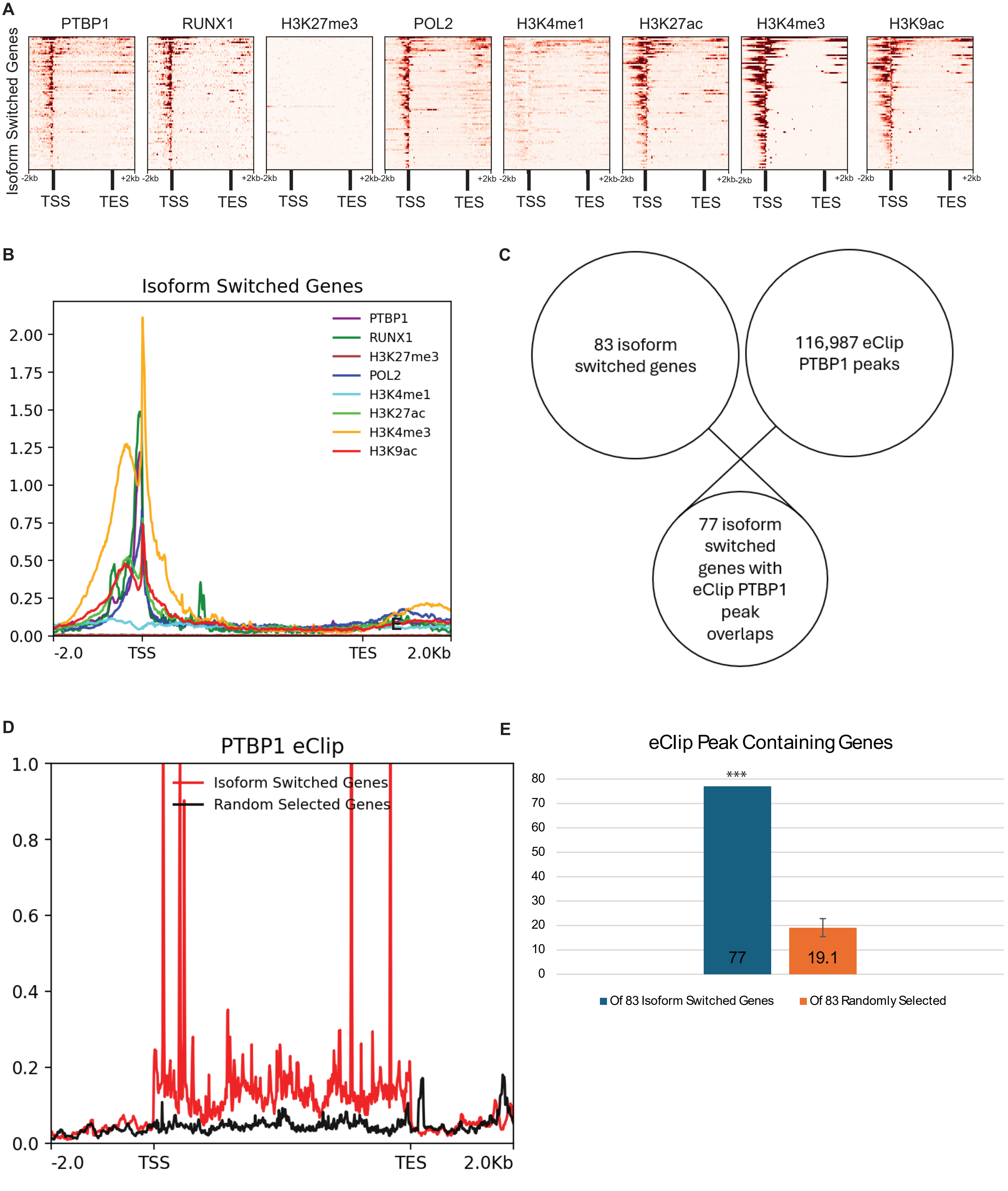

Supplementary fig 6

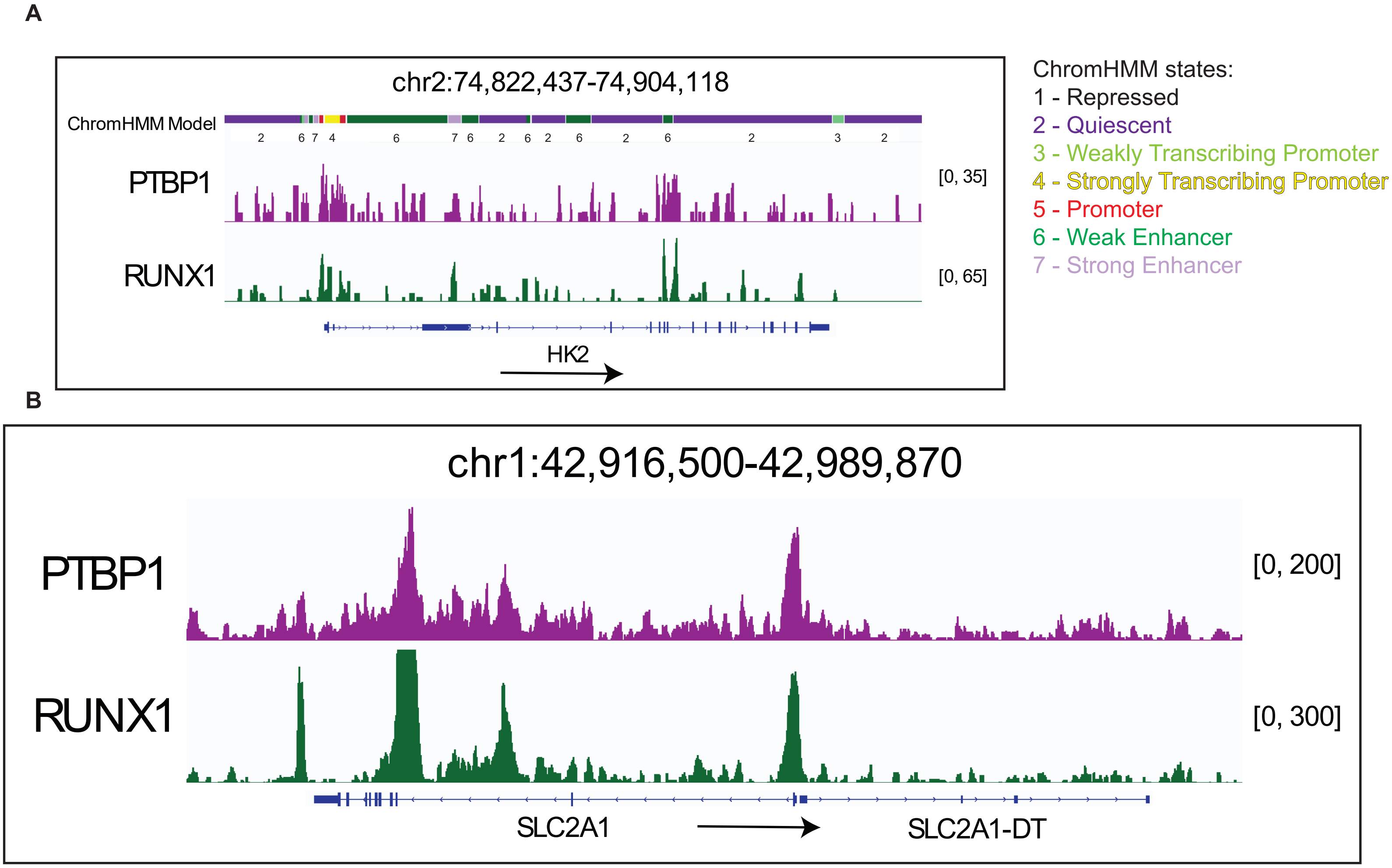

Supplementary Fig. 7

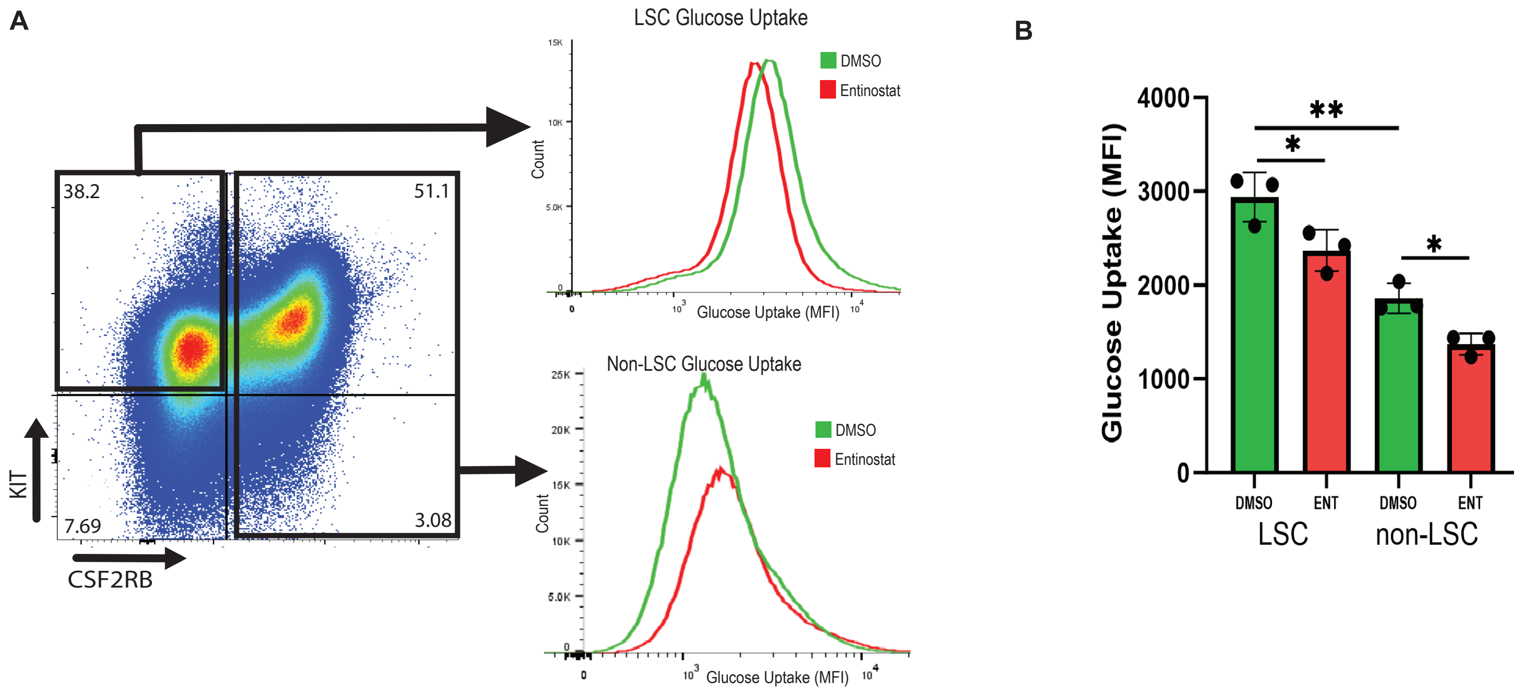

Supplement: Supplementary file 1 — Supplemental Material [file 41375_2025_2799_MOESM1_ESM.pdf]
